# Health Care Workers and Standard Precautions: Perceptions and Determinants of Compliance in the Emergency and Trauma Triage of a Tertiary Care Hospital in South India

**DOI:** 10.1155/2014/685072

**Published:** 2014-10-29

**Authors:** Sangini Punia, Suma Nair, Ranjitha S. Shetty

**Affiliations:** ^1^Department of Anesthesiology, University of Iowa Hospitals and Clinics, Iowa City, IA 52246, USA; ^2^Department of Community Medicine, Kasturba Medical College, Manipal University, Manipal, Udupi, Karnataka 576 104, India

## Abstract

*Background*. Careful adherence to standard precautions can protect both health care workers (HCWs) and patients from infections. The present study identified the perceptions and compliance with the use of standard precautions and assessed the determinants of noncompliance among the HCWs in an emergency and trauma triage centre. *Methods*. A cross-sectional study using a semistructured questionnaire was carried out to collect the relevant information from the study participants. *Results*. A total of 162 HCWs were recruited into the study, who reported varying degrees of compliance with standard precautions. While most of them declared the use of hand rub (95%) and gloves (77%), reported use of protective eye gear and outer protective clothing was very low (22 and 28%, resp.). Despite a perceived risk of exposure to blood-borne infections, 8% of the HCWs had not completed the hepatitis B vaccination schedule. About 17% reported at least one needle stick injury in the past year but only 5.6% received medical attention. *Conclusion*. Inadequate adherence to standard precautions among health care providers warrants new training and monitoring strategies. Establishment of an effective occupational health cell incorporating these elements including periodic surveillance could be the way forward.

## 1. Introduction

Standard precautions are the minimum infection prevention practices that apply to all patient care, irrespective of suspected or confirmed infection status of the patient, in any health care setting. These practices aim to both protect health care workers (HCWs) and prevent them from transmitting the infections to their patients. Standard precautions include hand hygiene, use of personal protective equipment (e.g., gloves, gowns, and masks), needle safety, and safe handling of potentially contaminated equipment or surfaces in the patient environment including respiratory hygiene (cough etiquette) and disposal of sharps, body fluids, and other clinical wastes properly [1–3].

Health care workers face the occupational risk of exposure to infection with blood-borne pathogen during the course of their routine work in the wards, intensive care units, emergency/trauma triage, and so forth. Worldwide, almost three million HCWs experience percutaneous exposure to blood-borne pathogens each year [4]. Despite infection control precautions and availability of hepatitis B vaccine, health care providers remain at risk of acquiring blood-borne infections [5]. Many exposures can be prevented by careful adherence to existing infection control precautions, immunization against hepatitis B, and provision of personal protective equipment during the management of emergencies [6–8].

Despite the availability of detailed guidelines, the knowledge and compliance with standard precautions vary among HCWs and have been found to be inadequate in both developed and developing countries [9–11].

Though there are reports regarding the compliance with standard precautions among HCWs in various parts of India, there is a paucity of information about the same among HCWs functioning in the emergency department of the health care settings. In this context the present study was undertaken to identify the perceptions and compliance with the use of standard precautions as well as to assess the determinants of noncompliance among HCWs in the emergency and trauma triage centre of a tertiary care hospital in southern Karnataka, India.

## 2. Methods

A cross-sectional questionnaire based study was carried out after obtaining ethical clearance from the institutional ethics committee (IEC). Study participants were the HCWs in the emergency and trauma triage centre of the hospital that has an active infection control committee and is compliant with biomedical waste management guidelines as well as good clinical practice. Participants included staff nurses, junior residents (interns and postgraduate trainees), and senior residents with a postgraduate training from the departments of medicine, surgery, and orthopaedics and who were frequently posted to the emergency and trauma triage at the hospital for emergency patient care. The staff nurses and junior and senior residents are the immediate care givers in cases requiring emergent care at this centre.

Estimating the practice of standard precautions among HCWs to be 52% [12] with a relative precision of 15% at 95% level of confidence, the minimum required sample size was 158. The total population of HCWs in the emergency and trauma triage centre at this hospital is 410. Accordingly a total of 162 participants were recruited using the population proportionate to size sampling technique. The participants were randomly recruited into the study, after obtaining a written informed consent. Confidentiality of the participants was maintained by giving them a coded identity.

A predesigned semistructured questionnaire was used to collect the relevant information. It was self-administered and included questions pertaining to compliance with the use of hand rub, personal protective gear, needle safety by HCWs during patient care, and perceptions of risk and barriers to compliance.

Data was collected over a period of two months, was tabulated and analysed using Statistical Package for Social Sciences (SPSS) version 15.0, and was presented in proportions.

## 3. Results

Of the 162 HCWs recruited into the study, 53 (32.7%) were staff nurses and 109 (67.2%) doctors. Of the doctors, junior residents comprising interns (52) and postgraduate trainees (45) represented more than half (59.8%) and the rest (7.4%) were senior residents.

Table 1 shows participants' compliance with standard precautions during patient care.

### 3.1. Hand Hygiene

Almost three-quarters (74.7%) of the study participants declared using hand rub as a personal protective measure and the majority (95.0%) claimed to use it after touching contaminated items. However, a little less than half (49.4%) stated its consistent use between patient interactions. This practice was reported highest among staff nurses (81.1%) and relatively less among junior residents (30.9%). Overall reported rates of hand rub use were high following procedures such as insertion of catheters (94.4%) and blood draws (85.7%). Sixty-three percent of the HCWs professed to always use hand rub after removing gloves.

### 3.2. Use of Personal Protective Equipment (PPE)

As per the reported data, use of gloves appeared to be considerable while drawing blood (81.0%) and during instances when coming in contact with mucous membranes or nonintact skin of the patients (88.3%).

About 45.6% of the participants admitted using face masks while suturing, another 53.1% while undertaking procedures like inserting a nasogastric tube, and 39.5% during a lumbar puncture. When confronted with a situation in which the risk of fluid splash was high and the HIV status of the patient was unknown, eye protection and protective gowns were said to be used by only 36 (22.2%) and 46 (28.4%) study participants, respectively. However, in situations where patient's HIV status was known to be positive, almost 96 (59.2%) participants stated the use of eye protection and gowns.

### 3.3. Needle Safety

Various unsafe practices reported by the HCWs with respect to using needles for patient care are depicted in Table 2. Effectively 59.3% of the respondents admitted to always recapping used needles, while another 30.0% reported the practice of disengaging needles manually from the syringe. Nearly 18% of the respondents confessed to not placing the used needles in designated sharps containers. Not surprisingly 17.2% of the HCWs reported a needle stick injury (NSI) at least once in the last twelve months.

### 3.4. Perception of Risk

More than 60% of health care workers considered themselves at high risk of getting exposed to blood-borne infections (64.2% for HIV and 61.7% for hepatitis B and hepatitis C infection). Despite this perception, 12 (7.4%) respondents had not received the complete schedule of the hepatitis B vaccine. Of the 17.2% respondents who claimed to have sustained a NSI in the past, only 5.6% admitted to reporting it to the concerned authorities (Table 3).

### 3.5. Barriers to Compliance

**Figure 1:**
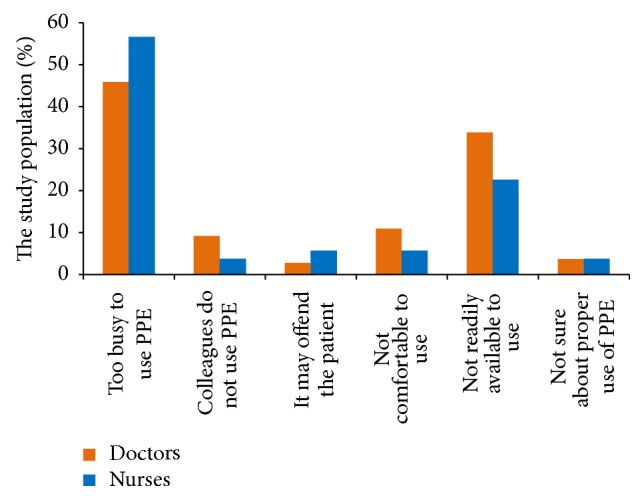
Barriers to PPE compliance among HCWs (*N* = 162).

The study participants expressed certain hindrances in complying with PPE when warranted (Figure 1). While lack of time appeared to be the most common reason, protective equipment not being readily available during an emergency situation also figured prominently as a deterring factor. Other less prominent reasons were the likelihood of offending patients and not knowing the correct method of using the equipment. Doctors seemed to be negatively influenced by their peers in not using PPE.

## 4. Discussion

The present study shows varying degrees of compliance with the different measures contained within standard precautions. The majority of the participants declared use of hand rub (74.7%) following most procedures. Compliance with glove use was reported by 85.1% in our study which corroborates the findings of a study from Nigeria [13]. The reported use was high (90%) when in contact with potentially infectious surfaces and this is in keeping with findings (97%) from a developed country setting [14].

In spite of the perceived risk of getting exposed to blood-borne infections, in the present study 7.4% of the HCWs had not completed the hepatitis B vaccine schedule. This is, however, better than the findings from studies conducted in the northern part of India [12] and the United States [15] that report higher rate of incomplete hepatitis B vaccination (23% and 25%, resp.).

Regarding eye protection, our study showed that only 22% reported compliance as compared to similar studies from India, where 32% of the HCWs wore eye protection when indicated [16, 17]. Contrary to this, compliance with the use of eye protective gear was found to be 63% in developed countries [14]. Likewise, in comparison to the developed countries [14] where a whopping 62% consistently used outer protective clothing, only about 28% of the respondents in this study claimed using it when indicated.

In the current study 17.2% of the HCWs reported at least one NSI in the previous one year, whereas a higher proportion of NSIs (30–57%) have been reported in studies from North West Ethiopia [18], Indianapolis [19], sub-Saharan countries [20], southern Ethiopia [21], and Indonesia [22]. Likewise, a study from North India [23] has also reported high proportion (80.1%) of NSIs among the HCWs of a tertiary care hospital. It is of concern that of the 17.3% NSIs reported in the study only 5.6% were conveyed to the concerned hospital authorities, which is in contrast to the findings (37.8%) from elsewhere [21]. It is also disturbing that more than half of the participants (59.3%) did not appear to follow needle safety precautions and recapping needles was recounted as a common practice. This is almost in sync with the findings from rural North India and Nigeria where about 30–40% of the participants resorted to recapping of needles always [16, 24]. Studies have shown 87% to 95% compliance with the correct disposal of sharps into designated sharps containers [14, 17], but the reported practice in this population is slightly lower (80%).

The study findings show the existence of inadequate needle safety precautions, low compliance with standard guidelines, and improper disposal of sharps among the health care workforce in a trauma care setting. This is despite the presence of an active infection control committee and the presence of posters stressing the need to comply with standard precautions.

There are certain inherent limitations in our study. As this report is based on self-reported cross-sectional study findings, we cannot rule out the possibility of bias resulting in an overestimation of the declared compliance. A longitudinal design could help us establish these findings with more certainty.

## 5. Conclusion

There is an urgent requirement to address the issues with reference to the barriers identified in the study. The postexposure prophylaxis policy of the health facility needs to be widely disseminated to the HCWs of the trauma triage centre for better reporting of NSIs. There is also the need to effectively put in place a hospital process that ensures ready availability of PPEs at the trauma triage centre. Besides, enhancement of the existing training on standard precautions for the trauma triage staff could reinforce the need to comply with standard guidelines however hard-pressed for time. Additionally, establishment of an effective occupational health cell incorporating all these elements including periodic surveillance could be the way forward. Future research could evaluate the efficacy of such an initiative in dealing with standard precautions and compliance.

## Figures and Tables

**Table 1:** Compliance with standard precautions among the participants (*N* = 162).

Study participants	Use of hand rub *n* (%)	Use of gloves *n* (%)	Use of masks *n* (%)	Use of eye gear *n* (%)	Use of gowns *n* (%)
Doctors (*N* = 109)	78 (71.5)	90 (82.6)	38 (34.8)	21 (19.2)	36 (33.0)
Nurses (*N* = 53)	43 (81.1)	48 (90.6)	36 (67.9)	15 (28.3)	10 (18.8)

Total (*N* = 162)	121 (74.7)	138 (85.1)	74 (45.6)	36 (22.2)	46 (28.4)

**Table 2:** Hazardous needle practices among the participants (*N* = 162).

Unsafe practices	Doctors (*N* = 109) *n* (%)	Nurses (*N* = 53) *n* (%)	Total (*N* = 162) *n* (%)
Recapping needles after use	79 (72.4)	17 (32.1)	96 (59.3)
Bending or breaking needles by hand after use	14 (12.8)	8 (15.1)	22 (13.6)
Manual removal of needles from syringes	26 (23.9)	23 (43.4)	49 (30.2)
Improper disposal of the used needles	25 (22.9)	4 (7.5)	29 (17.9)

**Table 3:** Perception and practice among HCWs (*N* = 162).

HCWs	Perceived risk of HIV/HCV infection *n* (%)	NSIs in the last one year *n* (%)	Received complete schedule of hepatitis B vaccine *n* (%)
Doctors (*N* = 109)	70 (64.2)	24 (22.0)	99 (90.8)
Nurses (*N* = 53)	34 (64.2)	04 (7.5)	51 (96.2)

Total (*N* = 162)	104 (64.2)	28 (17.2)	150 (92.6)
